# The Mini-Sigh Test: A New Haemodynamic Test of Fluid Responsiveness in Icu Patients Undergoing Pressure Support Ventilation

**DOI:** 10.1186/2197-425X-3-S1-A17

**Published:** 2015-10-01

**Authors:** A Messina, D Colombo, S Romagnoli, E Bonicolini, G De Mattei, F Longhini, AR De Gaudio, F Della Corte, P Navalesi

**Affiliations:** AOU Maggiore della Carità, Anesthesiology and Intensive Care, Novara, Italy; A.O.U Careggi - “Università degli Studi di Firenze”, Firenze, Italy; Ospedale S. Andrea, Vercelli, Italy; Università del Piemonte Orientale, Medicina Translazionale, Novara, Italy

## Introduction

Dynamic predictors of fluid responsiveness (FR) perform poorly in ICU patients receiving partial ventilatory assistance. Because these modes of partial support are increasingly used, FR dynamic indexes are applicable only in a few ICU patients [1]. To overcome these limitations, novel approaches for testing FR in ICU have been proposed, such as the passive leg raising and the end-expiratory occlusion. These tests, however, may not always be applicable [2]. During controlled mechanical ventilation, Pulse Pressure (PP) and left ventricle stroke volume are coupled; their variations are due to the reduction of right ventricle stroke volume consequent to ventilator insufflation and are either proportional to the tidal volume and closely related to preload dependence.

## Objective

We hypothesize that during Pressure Support (PS) a brief variation in intrathoracic pressure, such as that produced by a deeper inflation lasting some seconds, would differently affect PP in fluid responder and non-responders.

## Methods

**Figure 1:**
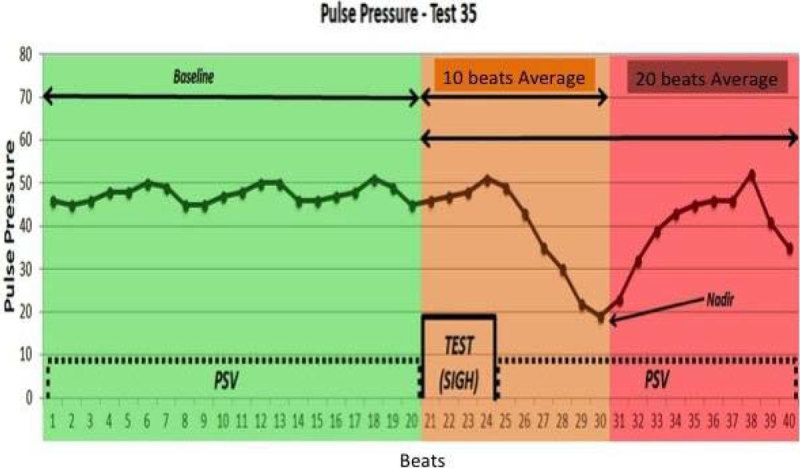
**ROC curves were utilized for comparing responders and non responders**

We investigated 30 ICU hemodynamically unstable patients undergoing PS. The fluid challenge consisted in 500 mL of Ringer Acetate in 10 minutes. Patients who showed an increase in CI ≥ 15% after fluid infusion were considered responders. Hemodynamic measurements were obtained through arterial waveform analysis by PRAM^®^. The ventilator was set adding to PS a time cycled (4 seconds) pressure-targeted (15, 25, and 35 cmH_2_0) sigh breath. The three preset levels of sigh pressure (SIGH_15; SIGH_25; SIGH_35) were applied in random order according to a pre-generated sequence. The PP variation (ΔPP) was calculated considering the average PP value in the 20 heartbeats preceding sigh application (baseline), and 10 (ΔPP_10) and (ΔPP_20) following the sigh. The lowest PP value obtained during the 20 heartbeats (ΔPP_Nadir) was compared with the baseline value (example of SIGH_35 in Figure 1).

## Results

There was no significant difference between ROC curves of responders and nonresponders with SIGH_15 and SIGH_25. The AUC of the ROC curve for ΔPP_Nadir with SIGH_35 was 0.87; a ΔPP_Nadir ≥ 35% predicted FR with sensitivity 76% and specificity 92%; finally, the AUC of ΔPP_Nadir was significantly greater than the AUC of ΔPP_10 (0.77; p = 0.03) and ΔPP_20 (0.75, p = 0.02) (Figure 2).

**Figure:**
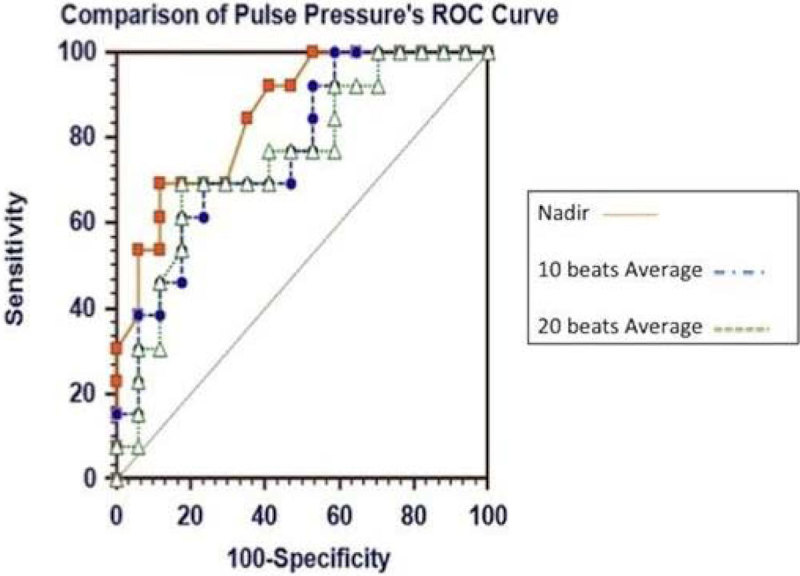
Figure 2

## Conclusions

In hemodynamically unstable patients undergoing PS, ΔPP_Nadir determined adding SIGH_35, while not SIGH_15 and SIGH_25, allows assessment of FR.
